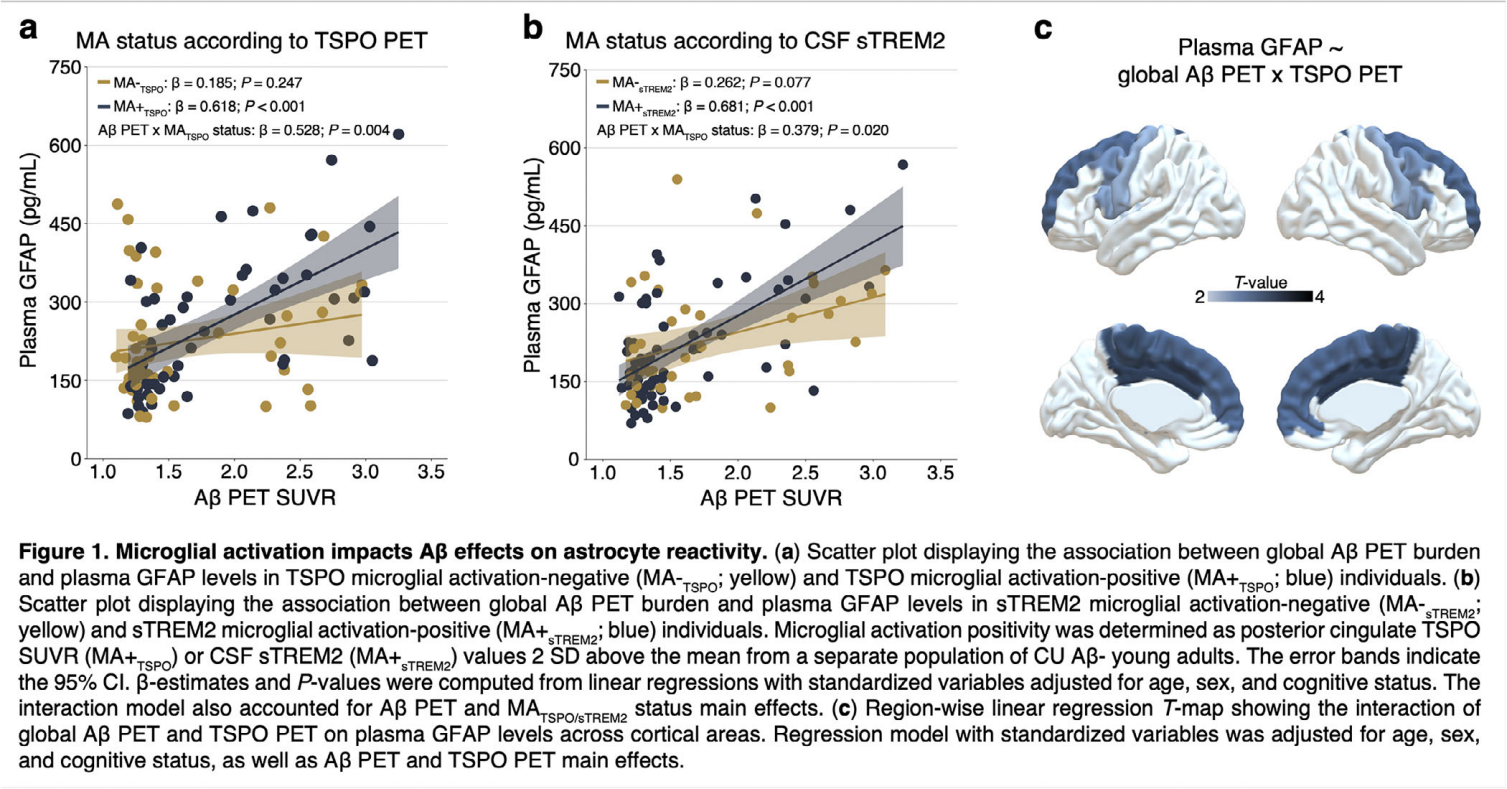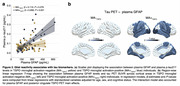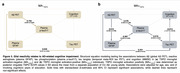# Microglia as a key player in Aβ‐related astrocyte reactivity

**DOI:** 10.1002/alz70856_102677

**Published:** 2025-12-25

**Authors:** João Pedro Ferrari‐Souza, Guilherme Povala, Nesrine Rahmouni, Bruna Bellaver, Pamela C.L. Ferreira, Douglas Teixeira Leffa, Firoza Z Lussier, Cristiano Aguzzoli, Wagner Scheeren Brum, Marco Antônio De Bastiani, Giovanna Carello‐Collar, Wyllians Vendramini Borelli, Joseph Therriault, Arthur C. Macedo, Stijn Servaes, Jenna Stevenson, Ilaria Pola, Serge Gauthier, Diogo O. Souza, Lucas Porcello Schilling, Mychael V. Lourenco, Gallen Triana‐Baltzer, Hartmuth C. Kolb, Andrea L. Benedet, Nicholas J. Ashton, Dana L Tudorascu, Henrik Zetterberg, Kaj Blennow, Tharick A Pascoal, Pedro Rosa‐Neto, Eduardo R. Zimmer

**Affiliations:** ^1^ Universidade Federal do Rio Grande do Sul, Porto Alegre, RS, Brazil; ^2^ University of Pittsburgh, Pittsburgh, PA, USA; ^3^ McGill University, Montreal, QC, Canada; ^4^ Brain Institute of Rio Grande do Sul (InsCer), Porto Alegre, Rio Grande do Sul, Brazil; ^5^ Graduate Program in Biological Sciences: Biochemistry, Universidade Federal do Rio Grande do Sul (UFRGS), Porto Alegre, Brazil; ^6^ Universidade Federal do Rio Grande do Sul, Porto Alegre, Rio Grande do Sul, Brazil; ^7^ University of Gothenburg, Gothenburg, Sweden; ^8^ Universidade Federal do Rio de Janeiro, Rio de Janeiro, Brazil; ^9^ Neuroscience Biomarkers, Johnson & Johnson Innovative Medicine, La Jolla, CA, USA; ^10^ Enigma Biomedical Group, Knoxville, TN, USA; ^11^ Department of Psychiatry and Neurochemistry, Institute of Neuroscience and Physiology, The Sahlgrenska Academy, University of Gothenburg, Mölndal, Sweden; ^12^ Banner Alzheimer's Institute and University of Arizona, Phoenix, AZ, USA; ^13^ University of Gothenburg, Mölndal, Sweden; ^14^ Federal University of Rio Grande do Sul (UFRGS), Porto Alegre, RS, Brazil

## Abstract

**Background:**

Glial reactivity has a major role in Alzheimer's disease (AD) etiology and progression, with astrocytes and microglia orchestrating neuroinflammatory responses. Even though experimental evidence indicate that activated microglia can induce astrocyte reactivity, it remains to be elucidated whether microglia activation influences amyloid‐β (Aβ) effects on astrocyte reactivity in the living AD human brain. Using imaging and fluid biomarker data in individuals across the aging and AD clinical spectrum, we tested the hypothesis that microglia influence the effects of Aβ pathology on astrocyte reactivity.

**Method:**

Data was obtained from the Translational Biomarkers in Aging and Dementia (TRIAD) study. We studied 62 cognitively unimpaired (CU), 26 mild cognitive impairment (MCI), and 13 AD dementia participants who had positron emission tomography (PET) data for TSPO microglial activation ([^11^C]PBR28) and Aβ plaques ([^18^F]AZD4694), as well as reactive astrocyte marker plasma glial fibrillary acidic protein (GFAP). We further assessed tau phosphorylation with plasma phosphorylated tau at threonine 217 (*p*‐tau217) and tau aggregation with [^18^F]MK‐6240 tau tangle PET. Additionally, a subset of 68 CU and 33 MCI individuals from the TRIAD cohort were evaluated with cerebrospinal fluid (CSF) microglial activation marker soluble triggering receptor expressed on myeloid cells 2 (sTREM2), [^18^F]AZD4694 Aβ PET, and plasma GFAP.

**Result:**

Regression analyses revealed that Aβ pathology was associated with astrocyte reactivity only in the presence of elevated levels of microglial activation, supporting that microglial activation influences Aβ effects on astrocyte reactivity. Similar results were observed when using TSPO PET and CSF sTREM2 to assess microglial activation (Figure 1). We also found that microglial activation and astrocyte reactivity were jointly associated with tau phosphorylation and aggregation (Figure 2). Importantly, the microglial‐dependent impact of Aβ on astrocyte reactivity contributed to cognitive impairment through tau pathology (Figure 3).

**Conclusion:**

Our results suggest that microglial activation is a major phenomenon linking Aβ and astrocyte reactivity in the living AD brain. These findings help to elucidate the intricate crosstalk between microglia and astrocytes in the AD brain, offering insights for the development of glia‐targeting therapies.